# Observations on Revaccination

**Published:** 1860-09

**Authors:** 


### OBSERVATIONS ON REVACCINATION.

BY MM. VLEMINCKX AND MARINUS.

M. Vleminckx detailed to the Belgian Academy of Medicine the results of the revaccinations which had been put into force at the prisons of Ghent and Vilvorde, the subjects together amounting to 1660. Of these, 379, or 16 per cent., were vaccinated with success. Of the 1660 there were in 716 manifest traces of a prior vaccination, and 471 exhibited marks of small-pox. Of the 716, 115, or 16 per cent., and of the 471, 220, or 46 per cent., were vaccinated with success. The author's conclusions, from these and other cases, which he has brought before the Academy on former occasions, are as follows:

1. Revaccination of subjects who have been well vaccinated, produces generally but very few useful effects.

2. Persons who have been the subjects of variola have much more cause to be revaccinated than those who have undergone proper vaccination.

3. Revaccination is successful in proportion to the length of time which has elapsed since the first vaccination or the attack of variola.

4. Until the age of twenty-five it is generally useless.

5. From that age to thirty-five it gives rise to useful results in a certain number of individuals, but this number is so extremely small, that, without proscribing it in such persons, it need not be warmly recommended to them.

6. After thirty-five it becomes a sure preservative, and consequently necessary.

7. Its failure at one period furnishes no reason for not having recourse to it at other epochs, as there is no reason to suppose that the receptivity may not return between the one and the other operation.

8. Revaccination of pupils in schools and colleges is useless, as is also the case with revaccination of soldiers in armies constituted as the Belgian.

M. Armieux also submitted to the Academy an account of revaccinations he had performed in the 25th regiment of the French line, stationed at Rome. The revaccinations amounted to 2021, of which number one-third succeeded. He found that when the virus was taken direct from the arm of healthy infants more than one-half the cases succeeded, while when it was taken from the arm of the adult, less than one-fourth of the cases succeeded. He feels convinced that the virus becomes enfeebled in energy in the adult, and is unsuited for the certain propagation of vaccination. He has also found that virus preserved under glass, or in glass tubes, to be of very uncertain efficacy, and sometimes quite inert. Contrary to most observers, he states that no age is refractory to re-vaccination, which succeeds equally well in youth as in riper age, his experience, however, with regard to the young being derived from a regiment, is necessarily very limited. Of the whole 2021 revaccinations, success was complete in 670, doubtful in 86, and wanting in 1254. With regard to prior vaccination, 1478 soldiers had been vaccinated in childhood, or for more than ten years, and had not suffered from variola. In these the revaccination succeeded in 505, 34 per cent., was doubtful in 62, and failed in 912. In 145 instances the subjects had not been vaccinated, but had had variola, and in these revaccination was successful in 58, doubtful in 4, and failed in 83. For six months after this revaccination, a considerable epidemic of variola prevailed in Rome, but throughout the regiment of more than 2000 men, only 8 cases of very benign variola, or varioloid, occurred. These 8 individuals had been successfully vaccinated in childhood; but, with the exception of one, they had either not been revaccinated or had been so without success.

M. Marinus observes that the conclusions M. Vleminckx arrives at in the paper we have referred to, are quite opposed to all former experience of vaccinators; and, without doubting the accuracy of the statements made, he thinks that until further investigations can be entered into they must be received as quite exceptional. It may be even worthy of consideration whether the prison life of the subject forming tke material of M. Vleminckx's experiments, have not favored receptivity at a more advanced age. In the mean time, awaiting the period when the question is to be brought before the Academy for full discussion, M. Marinus passes in review the various statistical proofs of the decay of the protective power of vaccination, and the efficacy of revaccination. The conclusions he comes to, from a great mass of figures, are:

1. That the preservative power of vaccination is of a true efficacy during the first ten years, and then goes on diminishing to the twenty-fifth or thirtieth year.

2. That the same law applies with respect to the preservation from variola by a first attack of this.

3. That the receptivity for a second vaccination is never greater than between the tenth and thirty-fifth year after the first, this being the period when revaccination has best succeeded, both in those who have been previously vaccinated, and in those who have had variola. This is, then, the period during which its performance is essential; and after this epoch of life has been passed, although revaccination may still prove useful, it need not be too warmly recommended.—*Medical Times and Gazette*, *Feb.* 8, 1860.

